# The utilization of critical care ultrasound to assess hemodynamics and lung pathology on ICU admission and the potential for predicting outcome

**DOI:** 10.1371/journal.pone.0182881

**Published:** 2017-08-14

**Authors:** Wanhong Yin, Yi Li, Xueying Zeng, Yao Qin, Dong Wang, Tongjuan Zou, Ling Su, Yan Kang

**Affiliations:** 1 Department of Critical Care Medicine, West China Hospital/West China School of Medicine, Sichuan University, Chengdu, Sichuan, People’s Republic of China; 2 Critical Care Medicine Department, National Institutes of Health, Bethesda, Maryland, United States of America; Nanjing University, CHINA

## Abstract

**Aim:**

Critical care ultrasound (CCUS) has been used by many Intensive Care Units(ICUs) worldwide, so as to guiding the diagnosis and the treatment. However, none of the publications currently systematically describe the utilization of CCUS to analyze the characteristics of hemodynamics and lung pathology upon the new admission to ICU and its potential role in patients’ prognosis prediction. In this retrospective clinical study, we have demonstrated and analyzed the characteristics of hemodynamics and lung pathology assessed by CCUS and investigated its potential to predict patient outcome.

**Methods:**

We have described and analyzed the epidemic characteristics of hemodynamics and lung pathology assessed by CCUS on ICU admission, which based on our database of 451 cases from the biggest medical center in Western China, between November 2014 and October 2015. The patients’ demographics, clinical characteristics, prognosis and ultrasonic pattern of hemodynamics and lung pathology had been analyzed. A bivariate logistic regression model was established to identify the correlation between the ultrasonic variables on admission and the ICU mortality.

**Results:**

The mean age of the 451 patients was 56.7±18.7 years; the mean APACHE II score was 19.0±7.9, the ICU mortality was 30.6%. Patients received CCUS examination of pericardial, right ventricle (RV) wall motion, left ventricle (LV) wall motion, LV systolic function, LV diastolic function, lung and volume of inferior vena cava (IVC) were 423(93.8%), 418(92.7%), 392(86.9%), 389(86.3%), 383(84.9%), 440(97.6%), 336(74.5%), respectively; The univariate analysis revealed that length of mechanical ventilation was significantly correlated with the diameter of IVC, tricuspid annular plane systolic excursion(TAPSE), mitral annular plane systolic excursion(MAPSE), early diastolic transmitral velocity to early mitral annulus diastolic velocity(E/e’) (p = 0.016, 0.011, 0.000, 0.049, respectively); The TAPSE, ejection fraction(EF), MAPSE, lung ultrasound score (LUS score) (p = 0.000, 0.028, 0.000, 0.011, respectively) were significantly related to ICU mortality. The multivariate analysis demonstrated that APACHE II, age, TAPSE, E/e’ are the independent risk factors for ICU mortality in our study.

**Conclusion:**

CCUS examination on ICU admission which performed by the experienced physician provide valuable information to assist the caregivers in understanding the comprehensive outlook of the characteristics of hemodynamics and lung pathology. Those key variables obtained by CCUS predict the possible prognosis of patients, hence deserve more attention in clinical decision making.

## Introduction

Focused Critical care ultrasound examinations (CCUS) in the critical care setting have been adopted widely and becoming an extension of the clinical critical care hemodynamic monitoring, lung pathology diagnosis and other organ function assessment because of their rapid, precise detection capabilities [1–9]. Previous studies have demonstrated that the ultrasounic evaluation at ICU admission could elevated the diagnostic accuracy and potentially improved healthcare quality [10–14].

Moreover, the highlight of critical care ultrasound examinations have the ability to visualize the pathological changes of the organs systematically, guiding the critical care physicians realize more details of the pathological pattern which could improve the supportive management accurately from several complicate underlying diseases [15–17]. In this aspects, the big data of the epidemic characteristics of ultrasound analysis of multi organ pathological dysfunction were required which would provide more evdiences for the clinical decision making and management. The relationship between hemodynamics, lung pathophysiological characteristics with variables of critical care ultrasonic examinations on ICU admission have not been elaborated systematically [18, 19], nor been identified whether any of the indicators could predict patient outcome.

In this manuscript, we have described and analyzed the epidemic characteristics of hemodynamics and lung pathology assessed by CCUS on ICU admission, which based on our database of 451 cases from the biggest medical center in Western China, Moreover, our investigation demonstrated that several ultrasonic indicator has the potential value to predict patient outcome.

## Methods

### Critical care ultrasound exam-on-admission database

The data was extracted from the CCUS exam-on-admission database in Western China Hospital in Sichuan University. This database has been created by the following elements: 1. The CCUS examinations were performed by the board certificated physician who has completed full CCUS training course and had more than half-year experience of critical care ultrasonic performance experience. Meanwhile, the results which diagnosed as “normal” or “abnormal” images have been reviewed and double-checked by other senior physicians. That means each patient have been scanned twice to get more accurate data if needed. Once the patients are showing the “abnormal” images, the pathologic examination was double checked with other physicians and pathologists immediately and then delivered to the attending/ senior physicians. Moreover, then the seniors decide to whether to change the management. 2. The Philips CX50 ultrasound system and Sonosite M-Turbo ultrasound system with an ordinary convex probe and an array probe were used for the data collection. 3. Patients were arranged to receive the echocardiography (Echo) and the lung ultrasound(LUS) examination within 12 hours after the admission. 4. Five different “points of Echo view” from which is possible to explore and obtain hemodynamic data, called subcostal long axis view(SLAX), subcostal inferior vena cava view(SIVC), parasternal long axis view(PLAX), parasternal short axis view(PSAX) and apical four chamber view(A4CH) [3, 20]. The Echo examination included the diameter of inferior cava vein(IVC), and the distensibility index of IVC(dIVC) when needed; measuring the pericardium effusion semiquantitatively; estimating the abnormal motion of the left ventricle(LV) wall; evaluating the right ventricle(RV), the LV diastolic function, the left atrium pressure, the systolic function and measuring the stroke volume. The lung ultrasound score (LUS score) exam was performed with the 12-region method, in which each side of the chest wall was divided into six regions [21, 22]. The LUS exam was required to identify the lung sliding, lung point, A-lines, B-lines, consolidation/atelectasis and pleural effusion. The physicians will decide which contents to be examined based on the image quality and the specific situation of the patient. All the CCUS assessments should be done within 30 minutes, and the findings were recorded automatically. The physician who did the CCUS assessment was also responsible for the patients’ clinical information collection on admission and follow up the outcome of the patients. All the data were entered into the database by the responsible physicians thereafter.

### Data collection and analysis

This project was designed as an retrospective clinical study and data analysis extracted from the 451 ICU admitted patients’ critical care ultrasound exam-on-admission information database, created by General Intensive Care Unit in the second biggest teaching hospital and research center in China from November 2014 to October 2015. The study focused on the patients’ hemodynamics and lung pathophysiological changes. Therefore, the ultrasonic variables which represented the volume status or volume responsiveness, the right heart function, diastolic and left heart, the systolic function and the lung ultrasound score which revealing the lung pathology were included. The other variables except listed above were excluded from this study. Two experienced attending physician double checked and recorded the data then analyzed as follows:(1) Characteristics of the ultrasonic pattern of hemodynamics: 1)the extent of the pericardial effusion which was semi quantitatively assessed in SLAX, and defined as follows [23, 24]: “Small” defined as posterior groove, depth<1cm and only in systole; “Moderate” defined as depth 1-2cm, posterior groove±elsewhere; “Large” defined as more than 2cm in depth and circumferential. 2) the regional abnormal motion of LV wall. 3) evaluation of RV [25]: eyeballing if RV is enlarged in A4CH and measure the area of the RV and LV. When the RV: LV area ratio >0.6 suggests “moderate” RV dilation, whereas a ratio >1.0 indicates “severe” RV dilation, the tricuspid annular plane systolic excursion(TAPSE) measuring is also required in this situation. 4) evaluation of LV systolic function [26–28]: eyeballing combined with measuring ejection fraction(EF) with modified Simpson’s rule and classify it as normal(EF>55%), mild dysfunction(EF 45–54%), moderate dysfunction (EF 30–44%) and severe dysfunction (EF <30%). 5) LV diastolic function [29–30]: assess and then classify into four categories with early diastolic transmitral velocity to late diastolic transmitral velocity ratio(E/A) and early mitral annulus diastolic velocity(e’). Left atrial pressure was evaluated with E/e’ combined with E/A, according to the EAE/ASE recommendations, which published in 2009[24]. 6) Volume status [20, 31, 32]: the diameter of IVC less than 1 cm or dIVC during controlled ventilation less than 12% indicates hypovolemia, the diameter of IVC more than 2cm indicates hypervolemia and dIVC during controlled ventilation more than 12% while diameter 1-2cm indicates normovolemia. Patients without controlled ventilation while diameter around 1-2cm was non-detectable whether the volume status were normal (recorded as unknown volume status). (2) Lung ultrasound pattern: identify the pneumothorax, A-lines, B-lines, consolidation/atelectasis and pleural effusion [4] and score the four LUS patterns in each exam region: presence of lung sliding with A lines or fewer than two isolated B-lines, scoring 0; multiple, well-defined B-lines (B1-lines), scoring 1; multiple coalescent B-lines (B2-lines), scoring 2; and the presence of a tissue pattern characterized by dynamic air bronchograms (lung consolidation), scoring 3. The worst ultrasound pattern has been observed in each zone were recorded to calculate as the sum of the score. The total score is 36 [21–22].

The study has been approved by the ethics committee of West China Hospital of Sichuan University; The waiver of the Requirements for Obtaining Informed Consent has been granted based on the observational nature of the study.

### Statistical analysis

The data were analyzed by using the SPSS22.0 statistical software. The measurement was expressed as a mean value ± standard deviation or median quartile (first–third quartile) according to their distribution for continuous variables, or as counts and percentages for categorical variables. Continuous variables were also expressed as ranges. The proportion of cases that accepted the examination for each aspect and the exclusive distribution of different pathological findings were described. A bivariate logistic regression model was established, univariate analysis was undertaken to identify the correlation between the ultrasonic variables of cardiorespiratory and ICU mortality. The multivariate analysis referred to the variables with a significant difference in univariate analysis and the variables without a significant difference but was supposed to be relative to the outcome by the physicians were undertaken to assess the independent risk factors for ICU mortality. p<0.05 was considered statistically significant.

## Results

### Demographic and clinical characteristic

The study included 451 cases that admitted to ICU with in one year period. As shown in Table 1, the mean age was 56.7±18.7 years, ranged from 11 to 97; The male to female ratio was 1.7:1, and the average APACHE II score was 19.0±7.9, ranged from 2 to 45. 388 of 451 patients (86.0%) were mechanically ventilated on PEEP of 3 to 28 cmH2O during their ICU stay, with the median time on ventilator support was 117(interquartile range [IQR], 28–299) hours. Upon ICU admission, the mean heart rate was 96.6±22.4 beats per minute, and the average mean arterial pressure(MAP) were 85.5±13.4 mmHg. In regards to the respiratory rate, the mean was 18.4±5.0, ranged from 9 to 46. The median length of ICU stay was 11days(IQR, 5–20). The total ICU and hospital mortality were 30.6% (138/451) and 31.5% (142/451), respectively. The diagnosis on the admission of the whole study group is presented in Table 2.

**Table 1:** Demographic and clinical characteristics on admission and the outcome of the studied subjects.

Variable	Measure	Range
**Gender (male/female)**	283/168 (1.7: 1)	Not available
**Age(year)**	56.7±18.7	11‑97
**APACHE II**	19.0±7.9	2‑45
**Heart rate(beats per minute)**	96.6±22.4	38‑178
**Systolic blood pressure(mmHg)**	121.5±20.4	53‑228
**Diastolic blood pressure(mmHg)**	67.7±13.2	23‑113
**Mean blood pressure(mmHg)**	85.5±13.4	35.7‑140
**Urine output per hour(ml)**	60(30, 100)	0‑500
**Respiratory rate(times per minute)**	18.4±5.0	9‑46
**PaO2/ FiO2**	204(142,286)	41.5‑901
**Ventilation/Non-ventilation**	388/63 (6.2: 1)	Not available
**PEEP(cmH2O)**	6.8±2.8	3‑28
**Length of mechanical ventilation(hours)**	117(28,299)	0‑2293
**ICU length of stay(day)**	11(5,20)	1‑89
**Hospital length of stay(day)**	20(11,34)	1‑402
**ICU mortality**	30.6%(138/451)	Not available
**Hospital mortality**	31.5%(142/451)	Not available

Values were expressed as mean ± standard deviation or median (interquartile range) and %(n/N), according to type of data and data distribution; abbreviations: APACHE II, Acute Physiology, Age, Chronic Health Evaluation II; PaO2, partial pressure of oxygen in arterial blood; FiO2, fraction of inspired oxygen; PEEP, positive end expiratory pressure.

**Table 2:** Admission diagnoses and the proportion.

Diagnosis	n = 451	%
**Respiratory disease**	95	21.1%
**Severe pneumonia**	34	7.5%
**AECOPD**	29	6.4%
**ARDS**	28	6.2%
**Others**	4	0.9%
**Shock**	77	17.1%
**Cardiac arrest**	19	4.2%
**Heart failure (acute /chronic)**	24	5.3%
**Renal failure(acute/chronic)**	11	2.4%
**Liver failure (acute /chronic)**	12	2.7%
**Acute obstructive suppurative cholangitis**	7	1.6%
**Severe acute pancreatitis**	44	9.8%
**Acute peritonitis**	16	3.5%
**Bowel obstruction**	5	1.1%
**Multiple trauma**	49	10.9%
**Tumor**	34	7.5%
**Stroke**	10	2.2%
**CNS infection**	13	2.9%
**Postoperative patients**	30	6.7%
**Organ Transplantation**	3	0.7%
**Burn**	2	0.4%

Values were expressed as number of cases and proportion; abbreviations: AECOPD, acute exacerbation of chronic obstructive pulmonary disease; ARDS, acute Respiratory Distress Syndrome; CNS, central nervous system.

### Ultrasonic pattern of hemodynamics and lung pathology of the cases on ICU admission

**Fig 1:**
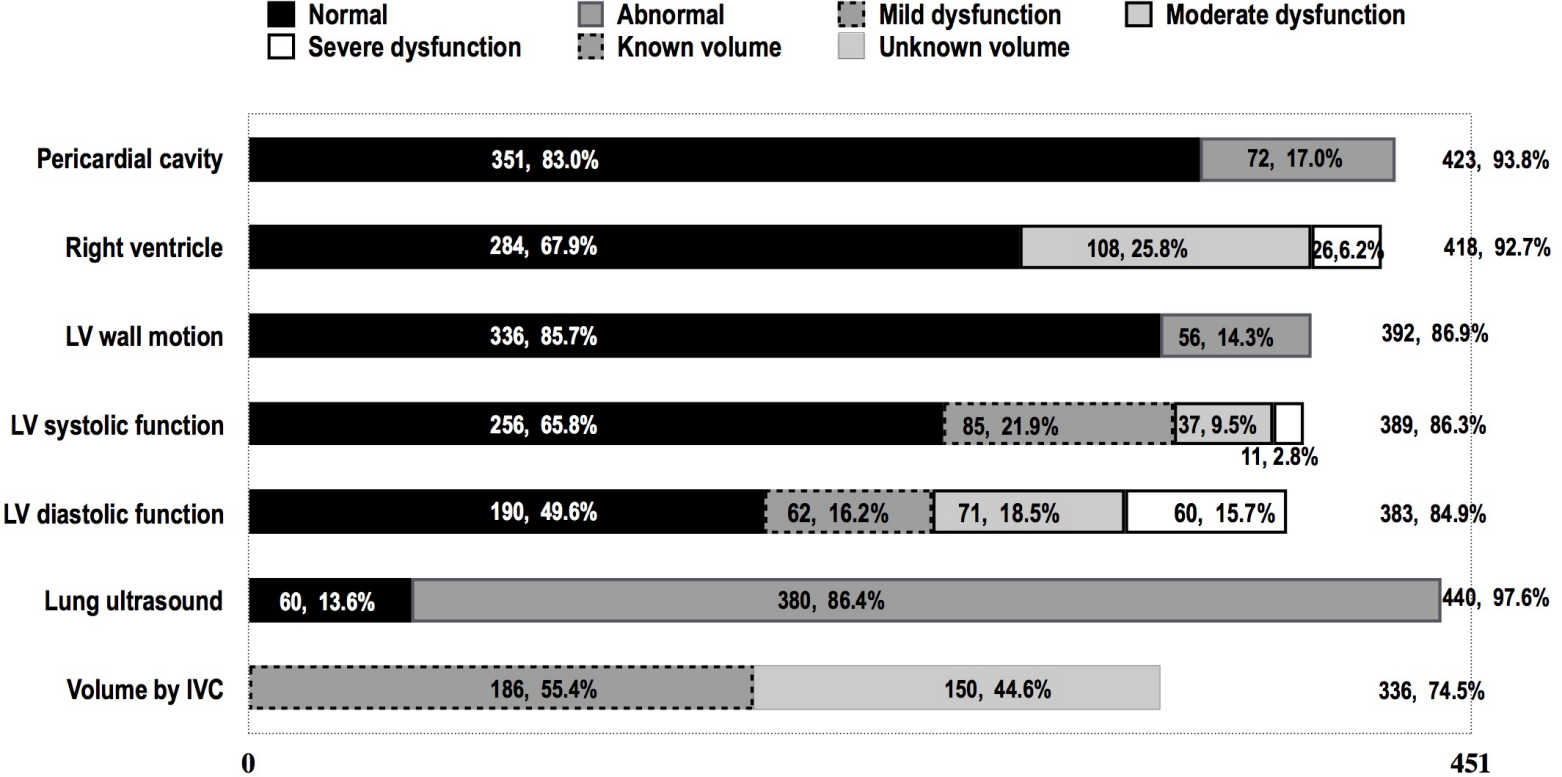
Completion proportion and findings of ultrasound examination on admission.

423 of 451 cases received the pericardial examination, the total number of pericardial effusion was 72(72/423, 17.0%). 418 cases received RV assessment, and 134 cases were considered as abnormal(134/418, 32.1%), in which the moderate and the severe abnormality were 108 cases (108/418, 25.8%), 26 cases (26/418, 6.2%), respectively; Regional abnormal motion of LV wall was detected in 56 cases out of 392 patients(56/392, 14.3%); LV systolic function was evaluated in 389 cases, and dysfunction was found in 133 cases(133/389, 34.2%), in which the mild, moderate and the severe dysfunction were 85 cases (85/389, 21.9%), 37 cases (37/389, 9.5%), 11 cases(11/389, 2.8%); 383 cases received diastolic function evaluation, and 193 cases were identified as abnormal, in which the mild, moderate and the severe dysfunction were 62 cases (62/383, 16.2%), 71 cases (71/383, 18.5%), 60 cases(60/383, 15.7%), respectively (Fig 1).

**Fig 2:**
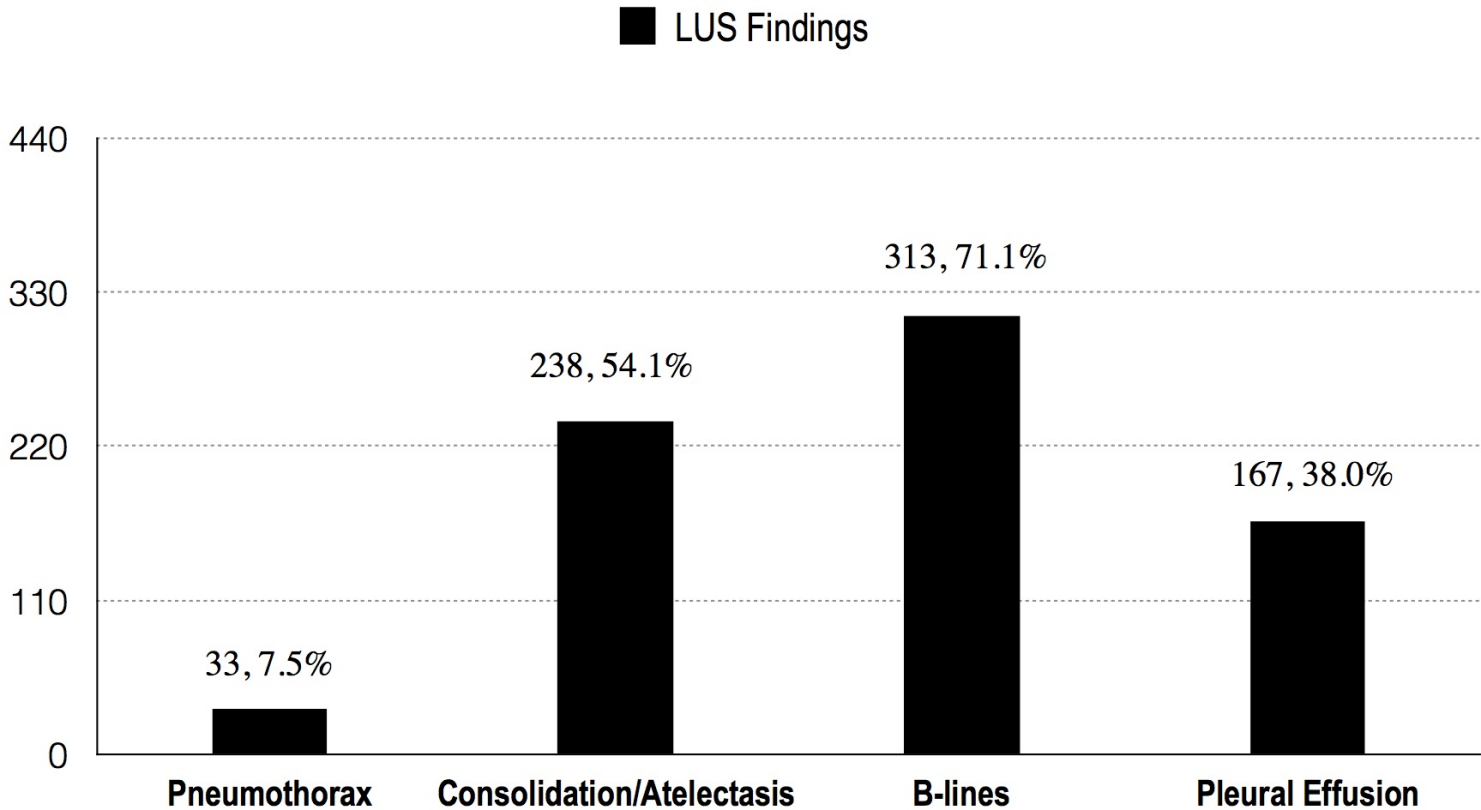
Abnormal findings in lung ultrasound examination on admission.

336 of 451 cases received the IVC exam. 186 cases had assess the volume status by examining IVC (186/336, 55.4%), which contains 53 cases of hypovolemia (53/186, 28.5%), 13 cases of normovolemia (13/186, 7.0%), 120 cases of hypervolemia (120/186, 64.5%); the rest 150 cases did not fulfill the criterion to assess the volume status by IVC examine individually (Unknown volume status, 150/336, 44.6%)(Fig 2).

**Fig 3:**
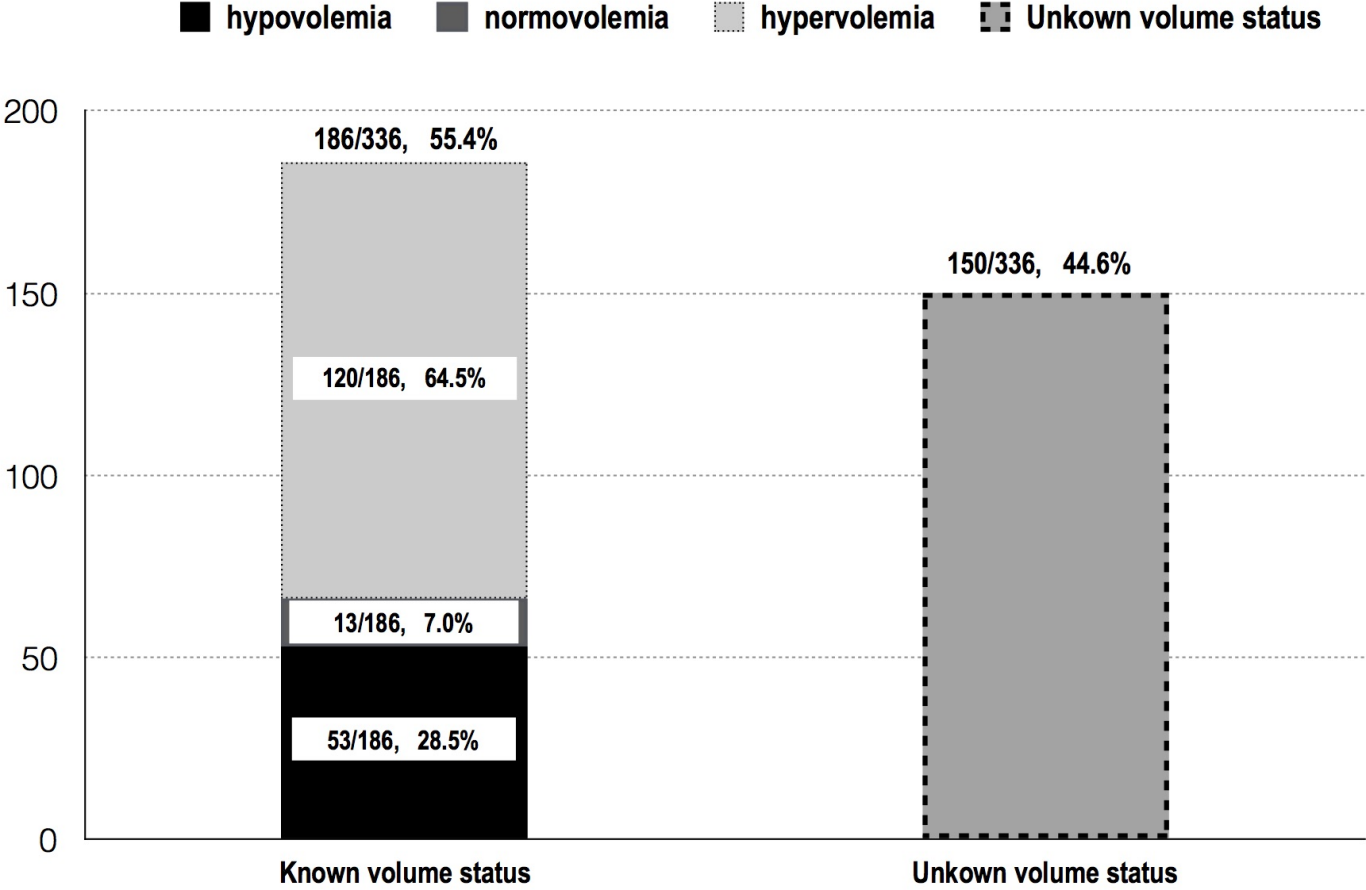
Volume status estimated by IVC examination on admission.

The lung ultrasound exam has been performed in 440 out of 451 patients. As shown in Fig 3, Positive lung pathology were identified in 380 cases (380/440, 86.4%), they are as follows: pneumothorax 33 cases (33/440,7.5%), consolidation/atelectasis 238 cases (238/440, 54.1%), B lines presences in 313 cases (313/440, 71.1%) and pleural effusion 167 cases(167/440, 38.0%).

### Prognosis analysis

The following ultrasonic variables such as volume status, RV, LV systolic function, LV diastolic function and LUS exam on admission has shown in Table 3. These variables were assessed in univariate correlation analysis, which revealed that length of mechanical ventilation was correlated with the diameter of IVC, TAPSE, MAPSE, E/e’ (p = 0.016, 0.011, 0.000, 0.049, respectively); and the TAPSE, EF, MAPSE, LUS score (p = 0.000, 0.028, 0.000, 0.011, respectively) were strongly associated with the ICU mortality (Table 4).

**Table 3:** Cardiorespiratory ultrasonic variables on admission.

Variable	Measure	Range	No. of Cases/N
**Diameter of IVC(cm)**	1.84±0.41	0.64‑2.72	336/451
**dIVC**	17.76(7.73, 29.14)	0‑140	121/451
**RV:LV Area Ratio**	0.56(0.42, 0.71)	0.13‑2.51	220/451
**TAPSE(cm)**	1.93±0.57	0.21‑3.52	323/451
**EF**	58.06±13.52	15.2‑84.8	302/451
**MAPSE(cm)**	1.40±0.55	0.26‑4.29	358/451
**E/e’**	7.72(6.17, 10.27)	0.12‑37.08	366/451
**SV(ml)**	60.59±20.20	14.40‑123.00	121/451
**LUS score**	12(6, 20)	0‑36	440/451

Values were expressed as mean ± standard deviation or median (interquartile range), according to type of data and data distribution; Abbreviations: IVC, Inferior Vena Cava; dIVC, distensibility index of Inferior Vena Cava; RV, right ventricle; LV, left ventricle; TAPSE, tricuspid annular plane systolic excursion; EF, ejection fraction; MAPSE, mitral annular plane systolic excursion; E/e’, early diastolic transmitral velocity to early mitral annulus diastolic velocity ratio; SV, stroke volume; LUS, lung ultrasound.

**Table 4:** Univariate correlation analysis: Correlation coefficients (r) and p values.

Indexes	Length of mechanical ventilation		ICU mortality	
	r	p	r	p
**Diameter of IVC**	0.133	0.016	0.252	0.372
**TAPSE**	-0.144	0.011	-1.063	0.000
**EF**	0.032	0.587	-0.020	0.028
**MAPSE**	-0.193	0.000	-0.994	0.000
**E/e’**	0.104	0.049	0.010	0.685
**LUS score**	0.049	0.314	0.029	0.011

Abbreviations: IVC, Inferior Vena Cava; TAPSE, tricuspid annular plane systolic excursion; EF, ejection fraction; MAPSE, mitral annular plane systolic excursion; E/e’, early diastolic transmitral velocity to early mitral annulus diastolic velocity ratio; LUS, lung ultrasound.

The multivariate analysis of the variables with a significant difference in univariate analysis and the variables which were chosen upon clinical concerns are the diameter of IVC, E/e', APACHE II, Gender, age, heart rate, MAP, urine output per hour, PaO2/FiO2 and respiratory rate. Among which the data analysis has shown that APACHE II, age, TAPSE, E/e’ were considered as the independent risk factors for ICU mortality, as shown in Table 5.

**Table 5:** Multivariate analysis between the cardiorespiratory ultrasonic variables and clinical indexes and ICU mortality.

Indexes	ICU mortality		
	OR	p	95% CI
**APACHE II**	1.077	0.014	1.015–1.142
**Age**	1.031	0.020	1.005–1.057
**TAPSE**	0.355	0.024	0.145–0.870
**E/e’**	0.903	0.023	0.827–0.986

Abbreviations: APACHE II, Acute Physiology, Age, Chronic Health Evaluation II; TAPSE, tricuspid annular plane systolic excursion; E/e’, early diastolic transmitral velocity to early mitral annulus diastolic velocity ratio.

## Discussion

In this study, we have indicated that there was a high completion rate for the CCUS exam on ICU admission, as the data shown in Fig 1, 97.6% cases received lung ultrasound examination, and the even volume status evaluation by IVC measurement had a completion rate of 74.5%. The exam covered all the contents that needed to be described the characteristics of the hemodynamics and the lung pathology. This study has demonstrated that it is feasible to complete the CCUS exam on ICU admission with in a short period (less than 30 minutes) to add more valuable information about the patient besides clinical characteristics, lab work, and other imaging exams. In the consideration of properties of noninvasive, responsible, rapid, affordable and reproducible data collecting at the bedside and providing specific data that may not be obtained from other diagnostic methods[33], we believe it is precious to encourage the CCUS exam to be utilized on admission in intensive care unit.

In term of the hemodynamics, our extracted data not only for the common ICU usage, such as the systolic function and volume status, also provide the parameters to detailing the heart structure and functions, such as diastolic function, RV function, pericardium and ventricular wall motion, etc. For instance, as data revealed in Fig 1, has included 17.0% of pericardial effusion, 32.1% of RV dysfunction, 14.3% of regional ventricular wall abnormal motion, 34.2% of LV systolic dysfunction and 50.4% of diastolic dysfunction. All the information above is crucial for patients who may need to have hemodynamic support treatment[25, 30, 34–39] The scattered indicators mentioned above may not be obtained from any other single equipment rather than CCUS exam, for example, PICCO and SWAN-GANZ, which both invasive and the usage are not suitable for all ICU patients [40–41]. For the LUS findings, the CCUS adds more valuable information than the single chest X-ray exam[42]. The Fig 1 and Fig 2 have provided the information that 86.4% the patients who received LUS exam had abnormal phenomenon, with which we have identified the pneumothorax (7.5%) and consolidation/atelectasis more accurately and rapidly, also we measured the lung water semi-quantitively with LUS score as well as discovered the distribution visually [43, 44].

Although this is a retrospective study and in current design, we haven’t focus onto discover the influence of the admission CCUS exam and apply to patients treatment, however, several studies have shown that the ultrasound exam has the ability to improved the diagnosticate accuracy and optimized the treatment [10–14, 45]. Nevertheless, we could adapt those similar researches and discoveries into our study and our utilized experiment design which based on the advanced and detailed CCUS exam. Moreover, by comparing with other monitoring or imaging equipments, the ultrasound device can visually focus on both heart and lung at the bedside, which highlights the valuable of the CCUS.

In this study, only 55.4% of the examined cases were clear to volume status(Fig 3). That’s might caused by the physicians selecting the diameter and the distensibility index of IVC to assess the volume. The former variable can only be used in the “extremely severe” situation(<1cm indicates severe hypovolemia while >2cm without variation in respiratory represents fluid overload) and the latter can only be employed in the situation of controlled ventilation when the tidal volume is more than 8-10ml/Kg [46]. Actually, there are many other methods in the position to evaluate the volume status and responsiveness, such as the respiratory variations of aortic blood flow, respiratory variations of common carotid artery blood flow, passive leg raising test, etc[47]. However, it was reasonable that they did not choose the parameters, since some of these variables were not well proved to be reliable by large sample trials,. In this setting, we have the amount of works to do when discussing the volume assessment by ultrasound. Moreover, CCUS has the competence to visually identify the status of fluid overload by IVC and LUS exam compare to others [48]. Fluid overload was significantly associated with higher mortality and morbidity among ICU patients, while discharge the excess fluid promptlymay contribute a better outcome [49, 50]. In this study, we found a high rate of fluid overload by IVC exam(64.5%, Fig 3) which is endorsed by a high presence of a percentage of B lines in lung ultrasound(71.1%, Fig 2).

Furthermore, our study showed that the CCUS on admission contribute to predicting the patient outcome. TAPSE, E/E’ are the independent risk factors as well as the APACHE II and age (Table 5). TAPSE responds for the right heart function [51, 52], while E/E’ represents the filling pressure of left atrium [53, 54]. Nevertheless, other parameters may contribute to a worse outcome, such as high LUS score, severe LV dysfunction, etc.[55, 56], However, our current study was not designed going to screen all the risk factors that predict ICU mortality.

This study also has couple limitations. First, being designed as a retrospective study, although we have two attendings to double check the data and identify the variables strictly according the standard and guidelines, the results still might be affected by the sampling error, and the prognosis analysis was not as credible as a prospective cohort study. Moreover, the objects of this study were extracted from the leading largest teaching hospital in Western China. The patients’ overall condition were severe (APACHE II 19.0±7.9), which might affect the representativeness of the study compare to the samples extracted from the the other hospitals or medical centers. The third, the high-quality bedside ultrasound needs well-trained physicians and guided by the well-defined protocol. Despite those limitations, this study has provided a significant sample of relevant information about the cardiorespiratory epidemic characteristic assessed by admission ultrasound exam, which showed a full sight of the ICU patients on admission and may be valuable for the clinical diagnosis and therapy plan decision making and subsequent design of clinical trials related to CCUS. A well designed prospective cohort study might be conducted to address those limitations we mentioned above.

## Conclusions

In conclusion, based on our study, CCUS exam on ICU admission which performed by experienced physicians can provide valuable variables, which independently related with patients’ outcome and helping physician to understand more about the perspective of the overall situation of the characteristics of hemodynamics and lung pathology. Hence, it merits more attention in clinical decision making.

## Supporting information

S1 Table Project database. (XLSX)
